# Phenotypic and Genetic Stability of the *Aldrovanda vesiculosa* L. Plants Regenerated in Tissue Culture

**DOI:** 10.3390/genes16091003

**Published:** 2025-08-25

**Authors:** Marzena Parzymies, Katarzyna Głębocka, Magdalena Pogorzelec, Barbara Banach-Albińska, Alicja Świstowska, Michał Arciszewski

**Affiliations:** 1 Institute of Horticultural Production, University of Life Sciences in Lublin, Głęboka 28, 20-612 Lublin, Poland; 2 Institute of Plant Genetics, Breeding and Biotechnology, University of Life Sciences in Lublin, Akademicka 13, 20-950 Lublin, Poland; 3 Department of Hydrobiology and Protection of Ecosystems, University of Life Sciences in Lublin, Dobrzańskiego 37, 20-262 Lublin, Poland; 4 Department of Botany and Plant Physiology, University of Life Sciences in Lublin, Akademicka 13, 20-950 Lublin, Poland

**Keywords:** micropropagation, molecular markers, somaclonal variation, true-to-typeness, waterwheel

## Abstract

Background: Tissue culture might be a method supplementing traditional plant propagation in various fields, like agriculture, medicine, industry, and the active conservation of plant species. For the purpose of plant restoration, it is important that the obtained progenies are identical with the mother plants to ensure the true-to-typeness of the future population. Methods: In the present study, the stability of *Aldrovanda vesiculosa* regenerants obtained in vitro through phenotypic and genetic analysis was estimated. Clones of aldrovanda plants were cultivated in tissue culture in the 1/10 MS liquid medium under the same conditions for over a year, with five weeks of subculturing. Results: It was observed that two clones formed plants that displayed atypical growth structures, the shoots were shorter with many lateral shoots, and they had a lower fresh weight. They also formed fewer and smaller snap-traps, which, in the case of carnivorous plants, determines the capability of catching prey. The 35 in vitro regenerated plants and 5 specimens obtained from the natural habitat were subjected to genetic analyses with two molecular markers: start codon targeted (SCoT) polymorphism and sequence-related amplified polymorphism (SRAP). Despite the visible morphological variants, the genetic stability of all the regenerants with the individuals from natural stands was confirmed. All of them were monomorphic except three bands that were obtained for reference, where individuals were amplified with SCoT28 and me12-em13 SRAP primers. Conclusions: As shown in the presented research, it might be recommended to use different methods to evaluate the stability of in vitro cultivated plants.

## 1. Introduction

The preservation of genetic diversity in plant populations has emerged as a pivotal concern in the realm of nature conservation. Particular emphasis has been placed on preserving the gene pools of populations threatened with extinction [1]. Ex situ conservation is recognized as a significant tool that can complement traditional in situ conservation efforts, particularly in cases involving species inhabiting unstable environments or those with limited reproductive capacity. In such cases, the employment of tissue culture techniques may serve as a method that fosters active protection or restoration of populations [2,3]. Presently, in vitro propagation has become a prevalent method for addressing the challenges associated with the preservation of the gene pool of rare and endangered plant species [4]. Sarasan et al. [3] indicate that micropropagation facilitates the expeditious mass propagation of endangered plant species and their long-term storage. The significance of integrating micropropagation and cryoconservation as a gene banking approach for rare genotypes is also underscored. As demonstrated by Reed et al. [5] and Pence [6], in vitro techniques facilitate the generation of a substantial number of specimens that are both healthy and genetically homogenous.

A few examples of the use of micropropagated carnivorous plants in conservation include *Drosera burmannii* [7], *Aldrovanda vesiculosa* [8], *Dionaea muscipula* [9], *Drosera rotundifolia* [10,11], and *Nepenthes khasiana* [12]. However, according to Caldeira et al. [13], although tissue culture is an important biotechnological tool for this group of plants, there is a lack of protocol for mass propagation of the species in tissue culture, and scientific information on the subject is presented at a slow pace.

As Barhill-Dilling [14] demonstrates, the progeny plants produced through micropropagation may be used for effective introduction or reintroduction of threatened and endangered plant species. Nevertheless, the fundamental concern associated with tissue culture is the genetic stability of the propagated plants. The term ‘somaclonal variation’ is used to describe the presence of phenotypic and DNA variation among plant clones. This phenomenon may be caused by the process of tissue culture, and consequently, the term ‘tissue culture-induced variation’ is sometimes employed to indicate the effect of environment [15]. Somaclonal variation is particularly pronounced in the case of indirect organogenesis or when high doses of growth regulators are used [15,16]. Therefore, it is recommended to use direct organogenesis methods, avoiding the prolonged subculturing and the monitoring of genotypes with molecular markers (RAPD, ISSR, and AFLP) or flow cytometry.

One of the species for which tissue culture propagation is applied and then reintroduction of the obtained progeny specimens in the natural habitat is undertaken for the means of active protection is *A. vesiculosa* L., the waterwheel plant. It is an aquatic carnivorous plant belonging to the *Drosearceae* family. It inhabits shallow, standing dystrophic waters across Europe, Asia, Australia, and Africa. It forms rootless shoots free-floating just below the water surface [17]. The leaves are arranged in characteristic whorls of 4–9, with a maximum diameter of 23 mm. Each fully developed leaf forms a trap which is composed of a two-lobed lamina with a midrib and 3–6 long bristles [18,19]. When mechanically irritated by prey, traps close rapidly. Bristles probably guide prey toward the trap, and they might prevent casual objects from entering the trap [20]. The length of the stem (6–20 cm) depends on various factors, both related to the abiotic environment (physical and chemical parameters of the water of solar radiation intensity), and biocenotic (availability of potential prey). The growth season lasts from early–mid-spring to early autumn. Temperate waterwheel populations flower and set seeds under optimal conditions; however, such observations are seldom documented [21]. The propagation of plants is predominantly vegetative, characterized by the formation of shoots and subsequent branching [17,22,23]. In the middle of autumn, in response to adverse conditions, plants form turions, which are a type of dormant overwintering buds that sink to the bottom of water reservoirs. Turions are formed by the dense leaves growing at highly shortened internodes that surround and protect the apex from freezing [23,24,25,26,27]. It has been shown that the overwintering phenomenon is associated with a significant decline in population size (turions are often carried ashore or freeze to death), and in many cases, the plant survival rate has been reported at 20–30%. In spring, the surviving turions float to the surface and shoots resume the growth cycle [28].

According to Adamec [23], Euro-Asian temperate populations are distinct from Australian ones. The former produce morphologically distinct and highly dormant winter buds (turions), and they do not contain the pigment anthocyanin. In contrast, the latter produce only non-dormant winter shoot apices and contain anthocyanin. European plants exhibit low rates of flowering and rarely produce viable seeds [28,29]. They primarily propagate through vegetative reproduction via apical branching of the shoots [17,25].

Despite its widespread presence on a global scale, the population of *A. vesiculosa* is present in isolated habitats. Legislative protections have been instituted for this species in all countries within its distribution. The conservation status of *A. vesiculosa* is of concern on both a global and regional scale. This concern is primarily due to the plant’s rarity, habitat specificity, and ongoing threats. According to the IUCN Red List, the species has been designated as endangered (EN) in accordance with criteria that include a restricted area of occupancy, significantly fragmented populations, and a persistent deterioration in habitat quality [30,31]. It is also listed in the Bern Convention in the Conservation of European Wildlife and Natural Habitats Appendix I as a species requiring specific habitat conservation measures [32]. In Poland, it is stated as critically endangered (CR) on the Polish Red List of pteridophytes and flowering plants [33].

Historically, *A. vesiculosa* has been found to inhabit all continents of the Old World. In contemporary times, it has been acknowledged as being in markedly low abundance and has become extinct in numerous regions and countries [30,34]. At least seventy-nine historical sites of the species were recorded in Poland in the last 200 years, but only nine natural sites were confirmed between 2006 and 2013 [35,36]. In the 1970s, the reintroduction of aldrovanda in select locations in Poland was met with documented success. The plants were obtained through the process of tissue culture [35]. According to the IUCN, such actions remain the most effective measures of conservation for this species [30].

The primary objective of this study was to investigate somaclonal variation among *A. vesiculosa* specimens cultivated in tissue culture for over one year, based on the morphological features and genetic analysis (SCoT and SRAP molecular markers). The results of this investigation will be instrumental in the reintroduction activities that are implemented for the purpose of ensuring the active protection of the species.

## 2. Materials and Methods

### 2.1. Tissue Culture Conditions and Morphological Observation

The plant material for tissue culture establishment was *A. vesiculosa* individuals collected from two peat bog lakes located in the Łęczna-Włodawa Lakeland in Eastern Poland. The geographical coordinates of the two lakes in question are as follows: Lake Łukie: 51°24’.40.30″ N and 23°04’56.73″ E; Lake Orchowe: 51°29’27.63″ N and 23°34’26.12″ E. The mother plants selected for in vitro propagation were characterized by typical growth for the species, in terms of length of shoot (10–15 cm). In the laboratory, the shoots were defoliated, cut into 2 cm pieces, and then surface-sterilized with sodium hypochlorite (NaOCl, Chempur, Piekary Śląskie, Poland) at a concentration of 0.25% for five minutes. The disinfected shoot fragments were then placed individually in tubes containing 10 mL of the liquid medium, which consisted of Murashige and Skoog (MS) [37] macro- and microelements supplemented with 0.1 mg·dm^−3^ thiamine (vit. B_1_, Sigma-Aldrich, Saint Louis, MO, USA), 0.5 mg·dm^−3^ pyridoxine (vit. B_6_, Sigma-Aldrich, Saint Louis, MO, USA), 0.5 mg·dm^−3^ niacine (vit. PP, Sigma-Aldrich, Saint Louis, MO, USA), 2.0 mg·dm^−3^ glycine (Sigma-Aldrich, Saint Louis, MO, USA), 100 mg·dm^−3^ myo-inositol (Sigma-Aldrich, Saint Louis, MO, USA), and 20 g·dm^−3^ sucrose (Chempur, Piekary Śląskie, Poland). All the components were diluted 10 times. The medium composition was selected on the basis of the previous research findings [38]. The medium’s pH was established at 5.5. The regenerating individuals were placed in a fresh medium at 5-week intervals. The plants were cultivated in 450 mL jars, with 10 plants per jar, containing 200 mL of the medium, and covered with semi-transparent plastic lids. Each clone (i.e., progeny plants obtained from a single mother plant) was cultivated separately. The explant types, media, and conditions were the same for all the cultivated shoots. After a year, the observations on the morphology of the regenerants were conducted on 20 randomly selected plants from each of four separate clones (propagated from different mother plants).

The obtained results of the measurements were subjected to the statistical analysis performed with Arstat software (University of Life Sciences in Lublin, Poland), using one-way or two-way ANOVAs for a one-factorial design. The significance between means was estimated with Tukey’s confidence intervals at a 5% level of significance.

### 2.2. SCoT and SRAP Genetic Analysis

To conduct the genetic analyses, a total of forty plants were taken, of which 35 were regenerants obtained from in vitro. As DNA from the parent plants was not available, 5 randomly selected specimens from the natural habitat were used. The isolation of DNA was performed in accordance with the procedure described by Palfavi et al. [39], with minor modifications. The concentration and purity of the DNA were measured with a Nanodrop ND-1000 spectrophotometer (Thermo Fisher Scientific, Waltham, MA, USA). Each sample was then diluted to a concentration of 20 ng/µL. Two molecular marker methods were applied: start codon targeted (SCoT) polymorphism [40] and sequence-related amplified polymorphism (SRAP) [41]. Ten SCoT primers and 15 SRAP primer combinations were screened to select the ones that amplified clear and scorable bands. Sequences of SCoT and SRAP primers that were used in this study are shown in Table 1 and Table 2, respectively.

The volume and reagent concentrations of PCR were consistent in both methods. The reaction mixture was composed of 10 µL of the following components: 1× PCR buffer (100 mM Tris-HCl, 500 mM KCl, and detergent) (Thermo Fisher Scientific, Waltham, MA, USA); 0.5 µg/µL BSA; 2 mM MgCl_2_; 200 µM of each dNTP; 600 nM of primer (one primer in the case of the SCoT method and two primers in the case of SRAP); 0.5 U of Taq DNA polymerase (Thermo Fisher Scientific, Waltham, MA, USA); and 40 ng of DNA template. The thermal profiles of reactions are described in Table 3.

The PCR products were separated on 1.5% agarose gels stained with ethidium bromide, and their sizes were determined by comparing them with a fragment length standard (Thermo Fisher Scientific, GeneRuler 100 bp DNA Ladder Plus).

## 3. Results and Discussion

The growth and regeneration of plants in tissue culture is an asexual process involving the mitotic division of cells. Therefore, it is expected that the regenerating plants will be phenotypically and genetically identical to the stock plants [42]. However, some or even all of the progenies may be different from the donor plants [43]. The true-to-typeness of plants cultivated in vitro can be evaluated through phenotypic, cytological, and molecular analysis [44]. In the presented research, the phenotypic and molecular analyses were carried out to estimate the stability of *A. vesiculosa* progenies obtained in tissue culture.

### 3.1. Phenotypic Variation

**Figure 1:**
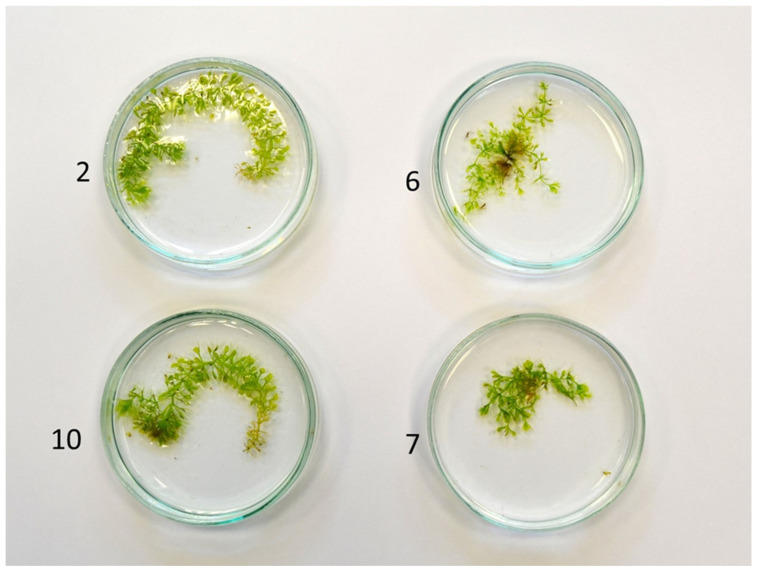
Morphology of the *A. vesiculosa* shoots propagated in vitro; clones 2 and 10—typical growth for the species; clones 6 and 7—branched growth.

During the cultivation, it was observed that two clones (marked as 6 and 7) formed shoots that displayed atypical growth characteristics. This phenomenon persisted throughout the entire cultivation period. The regenerated shoots were found to be shorter (5.07 and 4.76 on average, respectively), and they produced, on average, 7 and 4.7 lateral shoots of 2.2 and 2.54 mm (respectively) (Table 4). Two clones, marked 2 and 10, were selected from the remaining ones for the study as a reference, as they were characterized by typical growth for the species. Those clones produced longer shoots (10.63 and 10.28 on average), with fewer lateral shoots (1.60 and 2.30, respectively) that were formed at the top. Clones 6 and 7 shoots were also characterized by a lower fresh weight (17 and 7.89 mg, respectively) in comparison to clones 2 and 10 (31.67 and 31.53 mg, respectively). The morphology of the selected clones is illustrated in Figure 1.

**Figure 2:**
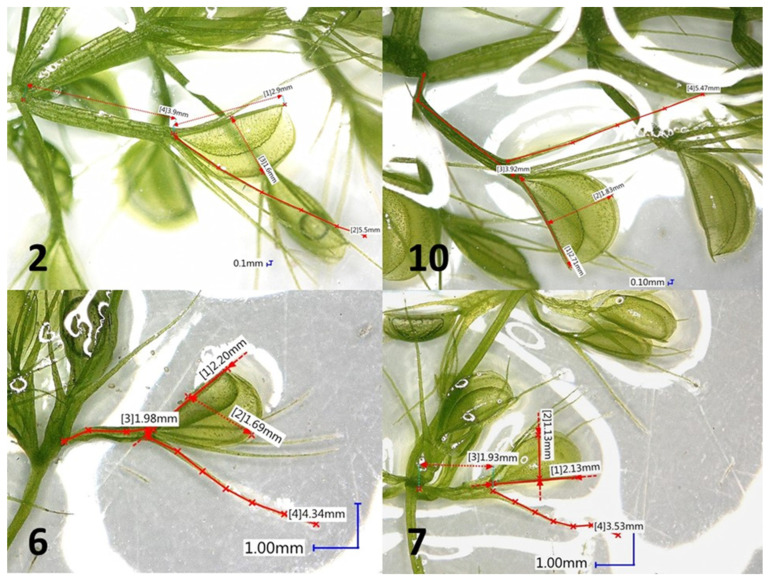
The appearance and measurements of the example traps of *A. vesiculosa* shoots propagated in vitro from different clones (2 and 10—typical growth for the species; 6 and 7—branched growth).

The *A. vesiculosa* is a carnivorous plant species with traps formed from two-lobed lamina with a midrib and long bristles. With regard to the capacity to capture prey, the structure and size of traps might be important features. Traps of natural plants range from 1.90 to 4.49 mm. Hortsmann et al. [45] studied the correlation between the trap size and prey type. However, due to partly digested prey in the traps, a reliable confirmation of that dependence was not possible. During in vitro cultivation, it was observed that the traps varied between the clones (Table 5). Significant differences in the number of whorls formed per centimeter of shoot were noted. Clones 2 and 10 formed significantly more whorls per 1 cm (3.0 both) when compared to clones 6 and 7 (2.3 and 2.1, respectively). The number of whorls is proportional to the number of traps. Furthermore, the size of traps (length and width) also varied between the clones. They were bigger in the case of clones 2 and 10 in comparison to clones 6 and 7. The appearance of traps is illustrated in Figure 2.

Morphological differences between plants can be easily detected on the basis of features such as plant structure, leaf morphology, or pigmentation [46]. They are usually detected during acclimatization or field cultivation [16]. The observed variations are usually genetically or environmentally induced [42]. According to Sharma et al. [47] and Karp [42], the direct formation of plant structures from axillary buds and shoot tips, without a callus phase, minimizes the risk of instability. The use of high doses of growth regulators, especially cytokinins, and increasing the number of subcultures and the culture age might also enhance the probability of somaclonal variation occurrence [16,48]. In the presented research, we can exclude any of the above factors that could influence the growth of plants. There are, however, some examples in the literature where more organized tissues, including meristem, presented somaclonal variation [16,42]. According to Podwyszyńska [49], the appearance of variants among lines cultured for the same time and under strictly identical culture conditions is apparently confusing.

### 3.2. SCoT and SRAP Genetic Analysis

Since the morphological changes in the case of two clones during cultivation in vitro were observed, we decided to perform molecular analysis to evaluate the genetic stability of *A. vesiculosa* regenerants obtained in tissue culture. SCoT and SRAP molecular markers were used in this research. Primers of both methods are designed in such a way that they tend to anneal to conserved regions of genes. In the case of SCoT, it is a region adjacent to the ATG start codon [40], and in the case of SRAP, CCGG (forward primer) and AATT (reverse primer) target exons and promoters or introns, respectively [41].

**Figure 3:**
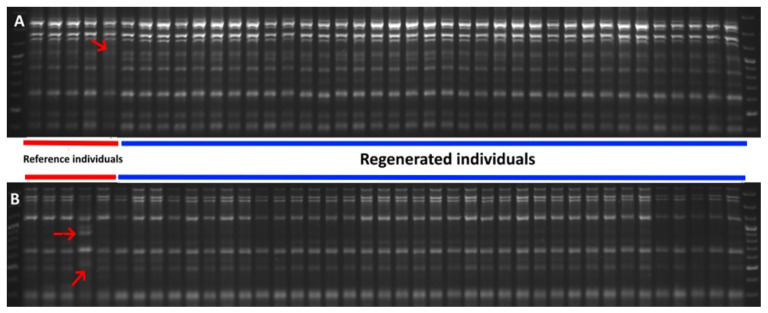
PCR products obtained for SCoT28 (**A**) and me12-em13 SRAP (**B**) molecular markers. Red arrows indicate polymorphic products obtained for two reference individuals. Red lines indicate reference plants and blue lines indicate in vitro regenerated ones.

In the present study, altogether, 40 PCR products were obtained: 15 were SCoT fragments and 25 were SRAP fragments. The sizes of SCoT products ranged from 500 to 2000 bp, and SRAP products ranged from 250 to 2500 bp. All of them were monomorphic except three bands that were obtained for reference individuals amplified with SCoT28 and me12-em13 SRAP primers (Figure 3A,B).

When in vitro regenerated individuals are analyzed by molecular markers, usually one mother plant and a few regenerants are enclosed, and monomorphic products are expected because they all should be genetically identical [50,51,52]. *A. vesiculosa* is generally considered a genetically monomorphic species [29,53,54,55]. In the presented paper, in the case of the five plants obtained from the natural stands, when amplified altogether with eight primers/primer pairs, only three polymorphic bands were obtained. Taking into consideration the genetic analysis only, all regenerated individuals were monomorphic as expected, which indicated that they genetically fit the *A. vesiculosa* profile.

The use of both methods, phenotypic and genetic, to evaluate the stability of *A. vesiculosa* tissue culture regenerants increased the reliability of somaclonal variant detection. According to Harding [44], morphological methods are more sensitive for assessing variation in individual plants, which could be confirmed in the present study, as there were no proven genetic variations between the plants regenerated in vitro, while the phenotypic changes were observed between the clones. The observed variation could not be connected with the external factors, such as the use of PGRs or culture age, as the differences in the growth structure were observed from the beginning of the cultivation. There is little data available demonstrating that tissue culture itself can affect the frequency and nature of somaclonal variation [56,57] or that it can arise from mutations already present in the donor plant [42].

In practice, micropropagation is often used in the protection of many endangered plant species, like *D. muscipula*, *Saussurea involucrate*, and *Orchid* spp. [58,59]. A combination of reintroduction and in vitro propagation is, at present, considered the most effective method for preserving the genetic resources of plant species that are threatened or endangered with extinction. However, it is often recommended that the confirmation of the genetic true-to-typeness of in vitro propagated plants should be proven, especially before reintroduction into natural stands [60,61,62,63].

We conclude that tissue culture is an effective method supplementing the traditional active protection of plant species. However, it needs an accurate approach, together with monitoring the stability and true-to-typeness of the regenerants. The monitoring usually includes a molecular marker analysis of the obtained plants. As shown in the presented paper, in the case of *A. vesiculosa* plants cultivated in tissue culture, the often-underestimated morphological observations might also detect occurring somaclonal variations. Any specimens that differ from standard plants for the species should be eliminated from further propagation and reintroduction into the natural habitat.

## Figures and Tables

**Table 1:** SCoT primer sequences.

Primer Name	Sequence (5′ 3′)
SCoT18	ACCATGGCTACCACCGCC
SCoT22	AACCATGGCTACCACCAC
SCoT28	CCATGGCTACCACCGCCA

**Table 2:** SRAP primer sequences and combinations applied in the study.

Forward Primer	Sequence	Reverse Primer	Sequence	Combinations Used
me2	TGAGTCCAAACCGGAGC	em2	GACTGCGTACGAATTTGC	me2-m2
me5	TGAGTCCAAACCGGAAG	em5	GACTGCGTACGAATTAAC	me5-em5
me10	TGAGTCCAAACCGGAAA	em11	GACTGCGTACGAATTCTA	me10-em12
me11	TGAGTCCAAACCGGAAC	em12	GACTGCGTACGAATTCTC	me11-em11
me12	TGAGTCCAAACCGGAGA	em13	GACTGCGTACGAATTCTG	me12-em13

**Table 3:** Thermal profiles of SCoT and SRAP methods.

Step	SCoT			SRAP		
	Temperature (°C)	Time (s)	Number of Cycles	Temperature (°C)	Time (s)	Number of Cycles
Initial denaturation	94	180		94	240	
Denaturation	94	60	35	94	60	5
Annealing	56	60		35	60	
Elongation	72	120		72	60	
Denaturation				94	60	36
Annealing				50	60	
Elongation				72	60	
Final elongation	72	300		72	420	

**Table 4:** Morphological features of four *Aldrovanda vesiculosa* clones propagated in vitro.

Clone Mark	Main Shoot Length (mm)	Number of Lateral Shoots/ Explant	Length of Lateral Shoots (mm)	Fresh Weight of Plants (mg)
2	10.63 A *	1.60 B	4.15 A	31.67 A
10	10.28 A	2.30 B	4.34 A	31.53 A
6	5.07 B	7.00 A	2.20 B	17.0 AB
7	4.76 B	4.70 AB	2.54 B	7.89 B

* Means followed by the same letter in columns do not differ significantly at *p* = 0.05.

**Table 5:** Morphological features of traps of *A. vesiculosa* plants propagated in vitro.

Clone Mark	No. of Whorls per 1 cm of Shoot	Length of Traps (mm)	Width of Traps (mm)	Length of Bristles (mm)	Length of Petioles (mm)
2	3.0 A *	2.77 A	1.71 A	5.67 A	3.88 A
10	3.0 A	2.98 A	1.75 A	5.64 A	3.82 A
6	2.3 B	1.93 B	1.36 B	4.09 B	1.86 B
7	2.10 B	2.11 B	1.17 B	3.61 B	1.95 B

* Means followed by the same letter in columns do not differ significantly at *p* = 0.05.

## Data Availability

All the required data related to the current study are included in this manuscript.

## Abbreviations

The following abbreviations are used in this manuscript:

MS	Murashige and Skoog medium (1962)
PGRs	Plant growth regulators
